# In-depth proteome analysis of brain tissue from Ewsr1 knockout mouse by multiplexed isobaric tandem mass tag labeling

**DOI:** 10.1038/s41598-023-42161-7

**Published:** 2023-09-14

**Authors:** Jin Woo Jung, Hyeyoon Kim, Joonho Park, Jongmin Woo, Eunji Jeon, Geeeun Lee, Minseo Park, Sarang Kim, Hoseok Seo, Seongmin Cheon, Kisoon Dan, Junghee Lee, Hoon Ryu, Dohyun Han

**Affiliations:** 1 Proteomics Core Facility, Biomedical Research Institute, Seoul National University Hospital, Seoul, 03082 South Korea; 2 Transdisciplinary Department of Medicine & Advanced Technology, Seoul National University Hospital, Seoul, 03082 South Korea; 3 Department of Pharmacology, CHA University College of Medicine, Pocheon-si, 11160 South Korea; 4 Center for Translational Biomedical Research, North Carolina Research Campus, University of North Carolina at Greensboro, Kannapolis, NC 28081 USA; 5 Department of Biomedical Sciences, Seoul National University Graduate School, Seoul, 03082 South Korea; 6 Interdisciplinary Program in Neuroscience, College of Natural Sciences, Seoul National University, Seoul, 08826 South Korea; 7 Boston University Alzheimer’s Disease Center and Department of Neurology, Boston University School of Medicine, Boston, MA 02118 USA; 8 Brain Science Institute, Korea Institute of Science and Technology (KIST), Seoul, 02792 South Korea; 9 Department of Medicine, College of Medicine, Seoul National University, Seoul, 03082 South Korea

**Keywords:** Proteomics, Proteomics, Molecular neuroscience

## Abstract

EWS RNA binding protein 1 (EWSR1) is a multifunctional protein whose epigenetic signatures contribute to the pathogenesis of various human diseases, such as neurodegenerative disorders, skin development, and tumorigenic processes. However, the specific cellular functions and physiological characteristics of EWSR1 remain unclear. In this study, we used quantitative mass spectrometry-based proteomics with tandem mass tag labeling to investigate the global proteome changes in brain tissue in Ewsr1 knockout and wild-type mice. From 9115 identified proteins, we selected 118 differentially expressed proteins, which is common to three quantitative data processing strategies including only protein level normalizations and spectrum-protein level normalization. Bioinformatics analysis of these common differentially expressed proteins revealed that proteins up-regulated in Ewsr1 knockout mouse are mostly related to the positive regulation of bone remodeling and inflammatory response. The down-regulated proteins were associated with the regulation of neurotransmitter levels or amino acid metabolic processes. Collectively, these findings provide insight into the physiological function and pathogenesis of EWSR1 on protein level. Better understanding of EWSR1 and its protein interactions will advance the field of clinical research into neuronal disorders. The mass spectrometry proteomics data have been deposited to the ProteomeXchange Consortium via the PRIDE partner repository with the dataset identifier PXD026994.

## Introduction

EWSR1 [Ewing’s sarcoma (EWS) RNA-binding protein 1/Ewing Sarcoma Break Point Region 1] is an RNA/DNA binding protein involved in various cellular process^1^ that plays diverse roles in different pathologies. For instance, it is associated with EWS, a highly carcinogenic and metastatic tumor that develops in the bone, such as the pelvis, rib, or spine, or related soft tissue, and spreads to the bone marrow. The disease is characterized by a morphologically heterogeneous tumor, mainly caused by chromosomal aberrations in the *EWSR1* and other genes^2,3^. Chromosomal translocation of *EWSR1* leads to the formation of the EWSR1/FEV (protein FEV) fusion protein with potential oncogenic features, which acts as a transcriptional repressor^4,5^. It also causes angiomatoid fibrous histiocytoma (AFH), an infrequent soft tissue tumor with low metastasis, which usually occurs in the subcutaneous region in children and adolescents^6^. A molecular indication of AFH is not well understood, with only specific gene fusion cases reported recently. Among them, the fusion of the gene for the cyclic AMP-responsive element-binding protein 1 (CREB1) with *EWSR1* is the most frequent genetic abnormality in the disease. It is possible that EWSR1 acts as a transcription activator Further, *EWSR1/ATF1* (the cyclic AMP-dependent transcription factor ATF-1) fusion gene was discovered by RT-PCR in tumor tissue derived from a patient with AFH, and it also discovered a function of constitutive transcriptional activator^7,8^. On the other hand, the EWSR1 protein has multiple functions. It participates in mitochondrial homeostasis by leading to ubiquitination and proteolysis of peroxisome proliferator-activated receptor γ coactivator (PGC-1α), a key protein involved in mitochondrial biogenesis, while maintaining its stability^9^. The *Ewsr*1 homozygous knockout (KO) (–/–) mice are born relatively smaller than wild-type (WT) mice, with a low survival rate, and with a diminished spleen and thymus size^10^.

Ewsr1 plays an essential role in neurodegenerative or brain disorders. Missense mutations of *EWSR1* in the central nervous system (CNS) are related to neurodegenerative diseases, such as amyotrophic lateral sclerosis. The neuronal nucleus of *Ewsr1* KO mouse in the motor cortex is significantly smaller than that in WT mouse, which causes an increase in the forelimb and hindlimb movements^11^. Another study reported on the role of *Ewsr1* in zebrafish embryonic development, investigated using morpholino injections^12^. In the zebrafish model, morphological desertions in the CNS in *Ewsr1*-deficient animals are accompanied by apoptotic cell death^12^. Likewise, EWSR1 plays a major role in diverse biological processes by interacting with other proteins, and therefore, it may adopt an exclusive molecular role in specific pathologies. Based on one microRNA study, *Ewsr1* deficiency leads to an increase in the levels of Mir125a- and Mir351-, which modulate the inhibition of autophagy^1^. However, most of these findings are based on genomics approaches or provide only partial proteomic information, e.g., on the proteins interacting with EWSR1.

Mass spectrometry (MS)-based proteomics allow for a highly accurate analysis of a cellular or tissue proteome, with various recent developments in the field facilitating specific protein discovery. For instance, protein identification efficiency can be increased and sample loss minimized by using filter-aided sample preparation (FASP) digestion^13^ and high-pH fractionation with high-performance liquid chromatography (HPLC)^14^. Further, multiplexed isobaric tandem mass tags (TMT), which contain *N*-hydroxysuccinimide (NHS) functional amine groups for labeling of the N-terminus of peptides, greatly improve the identification depth and throughput of MS-based proteomics. During the TMT analysis step, each isobaric label consists of different numbers of heavy isotopes within the reporter region and yields a unique reporter mass after fragmentation during tandem MS (MS/MS). This allows simultaneous identification and quantification of thousands of proteins^15^.

In the current study, we used TMT-labeling MS to investigate the specific molecular role of EWSR1. We analyzed the expression changes in the global proteome in the brain tissue of *Ewsr1* KO mice, and reconstructed the corresponding physiological networks and related disease mechanisms. These findings will shed light on EWS and other diseases.

## Experimental section

### Generation of Ewsr1 KO mice

Homozygous *Ewsr1* KO mice were generated as described by Agah et al.^16^. The targeting *Ewsr1* KO allele was injected into embryonic stem (ES) cells of TC-1 mice. Homologous recombination was confirmed by PCR and Southern blotting. Positive ES cells were inserted into C57BL/6J blastocysts, and the chimeras were bred with Black Swiss females (Taconic) to create F1 heterozygotes^11^. To obtain homozygotes, male heterozygotes were cultured in female heterozygotes^11^. PCR analysis of tail DNA using two sets of primers, WT (411-bp PCR product; 5′-TGG ATC CTA TGG ATC CTA CAG CCA GGC TCC-3′ and 5′-TGC TCG CTA GTG CTC TGT GAG CAG GAC-3′) and mutant (237-bp PCR product; 5′-TGG ATC CTA CAG CCA AGC TCC-3′ and 5′-CCT GTA TGA GTC CTG GTG TGG GTC-3′)^11^. All procedures regarding animal care and handling were approved and supervised by the Institutional Animal Care and Use Committee at KIST (Seoul, Korea), and all experiments were performed in accordance with relevant guidelines and regulations. This study is reported in accordance with ARRIVE guidelines^17^.

### Histological process

Six mice [three homozygous *Ewsr1* KO (−/−) mice, and three WT littermate controls) were transcardially perfused at 3 postnatal weeks old with 4% (v/v) buffered paraformaldehyde. The brains were extracted and post-fixed overnight. Those weights were measured and cryoprotected in 30% sucrose in PBS. After that, the brains were serially cryosectioned at either coronal or sagittal planes. Each section was stained with eosin or cresyl violet, and hematoxylin^11^.

### Brain tissue lysis

The extracted brain tissues of *Ewsr1* KO and WT mice were transferred to tubes with an approximately 5× greater volume of lysis buffer [4% sodium dodecyl sulfate (SDS), 2 mM Tris(2-carboxyethyl) phosphine, and 0.1 M Tris–HCl (pH 7.5) in HPLC-grade water]. The lysed brain tissue was immediately homogenized using Q700 sonicator (QSONICA, CT, USA). After homogenization, all samples were heated in a heating block at 95 °C for 1 h for protein extraction and delipidation. The extracted proteins were additionally filtered with Costar Spin-X 0.22 µm centrifuge tube filter (Corning Inc., NY, USA) to remove the remaining lipids. The protein concentration was determined by tryptophan fluorescence (WF) assay^18^. To remove contaminants and lipid contents from brain tissue lysate, 200 μg of protein was precipitated with acetone^19^. Briefly, approximately five sample volumes of cooled (− 20 °C) acetone were added, the samples mixed by gentle tapping, and stored in LoBind tubes (Eppendorf, Hamburg, Germany) at − 20 °C overnight. The samples were then centrifuged for 10 min at 15,000 rpm at 4 °C. The supernatant was discarded, and the above steps repeated. After drying at 25 °C for 15–30 min, the supernatant was completely evaporated, and the precipitated samples stored at − 80 °C until FASP digestion.

### FASP digestion

The protein extracted from the brain tissue was prepared as described in “Experimental section” and submitted to FASP digestion^20,21^. Briefly, the precipitated proteins were suspended in 40 μL of SDS buffer [2% SDS, 50 mM CAA, 10 mM Tris(2-carboxyethyl) phosphine, and 0.1 M Tris–HCl (pH 8.5) in HPLC-grade water] and vortex-mixed for approximately 3 min to completely dissolve the precipitates. The samples were denatured at 95 °C for 20 min and table top centrifuged. The samples were then mixed with 300 μL of 8 M urea that had been filtered through 0.22-μm Minisart filter (Satorious, Gottingen, Germany). The samples (approximately 350 μL) were then transferred to a 30 kDa Amicon ultra-0.5 mL centrifugal filter device (Amicon Ultra, Millipore, Ireland) and centrifuged at 14,000×*g* for 20 min. Urea solution (300 μL) was added once again, and the above steps were repeated. Then, the filtered sample was washed three times with 350 μL of 50 mM 4-(2-hydroxyethyl)-1-piperazineethanesulfonic acid (HEPES), and centrifuged at 14,000×*g* for 20 min. After discarded flow-through, an MS-grade trypsin/lys-C mix (Promega, Madison, WI, USA) was added at a 1:100 ratio (enzyme: sample; w/v) for the first digestion, and the samples incubated at 37 °C overnight. The digested peptides were then eluted by centrifugation at 14,000×*g* for 20 min. The second digestion was performed using sequencing-grade modified trypsin (Promega), added at a 1:1000 ratio (enzyme: sample; w/v), at 37 °C for 3 h. The peptides were then eluted by centrifugation at 14,000×*g* for 20 min. Sodium chloride (50 μL) was added to a 30 kDa filter to quench the reaction and about 350 μL was collected after centrifugation at 14,000×*g* for 10 min.

### TMT labeling

The concentration of peptides eluted after FASP digestion was measured using the WF assay^18^. TMTsixplex™ Isobaric Label Reagent Set was used with the manufacturer’s protocol^15^, with minor modifications. Each TMT reagent (0.8 mg) was dissolved in 120 μL of 100% acetonitrile, and 20 μL of the dissolved reagent was mixed with a corresponding volume of 25 μg peptides. Each sample (brain tissues from three WT mice and three *Ewsr1* KO mice) was individually labeled with a 6-plex TMT kit reagent (Thermo Fisher Scientific, Waltham, MA, USA). As a global internal standard, 10 μL of ovalbumin solution (0.26 μg/μL) was added to each sample, with enough acetonitrile for a final concentration of approximately 30% of acetonitrile (v/v). After incubation for 1 h at 25 °C, the labeling reaction was quenched by adding hydroxylamine for a final concentration of 5% for 15 min and all samples were pooled. The pooled sample was completely dried and desalted using an Oasis HLB column (Waters, Milford, MA, USA). The dried samples were stored at − 80 °C until use.

### Offline high-pH peptide fractionation

The pooled TMT-labeled sample was fractionated using an Agilent 1260 HPLC system (Agilent, Santa Clara, CA, USA) to reduce sample complexity. The sample was dissolved in 90 μL of buffer A (10 mM ammonium formate, pH 10, and 2% acetonitrile) and loaded onto a ZORBAX 300 Extend-C18 column (Agilent). Twenty-four fractions were collected by gradient elution with buffer B (15 mM ammonium formate, pH 10, and 90% acetonitrile), by increasing the amount of buffer B from 5 to 40% in 40 min and 20 min wash with 100% buffer B. The total run time was 1 h. The obtained fractions were completely dried and stored at − 80 °C until MS analysis.

### LC–MS/MS analysis

The dried samples were re-suspended in 50 μL of solvent A (0.1% formic acid in HPLC-grade water) for MS analysis. The samples were analyzed using a Q-Exactive Plus mass spectrometer (Thermo Fisher Scientific) coupled with an Ultimate 3000 UHPLC system (Thermo Fisher Scientific). Each sample (10 μL) was loaded onto a trap column (PEPMAP10 C18, 5 μm, 0.3 × 5 mm, Thermo Fisher Scientific) and the peptides were separated on an analytical column (EASY-Spray C18, 75 μm I.D. × 50 cm long, 2 μm, Thermo Fisher Scientific). The sample was analyzed using a 220 min gradient from 8% solvent B (0.1% formic acid in acetonitrile) to 60% solvent B at 300 nL/min. The following LC and MS parameters were used for analysis: a 2.0 kV spray voltage in positive ion mode and a capillary temperature of 320 °C. The MS scan range was set to 350–1800 m/z with 70,000 resolution at m/z 200 for the precursor ion. An automatic gain control (AGC) target of the full MS was 3E6 with a maximum injection time (IT) of 20 ms. The MS/MS scan resolution was set at 35,000. For MS/MS, up to 15 peptide precursors were isolated for HCD fragmentation (isolation width of 0.7 Th, AGC target value of 2E5, and the maximum IT of 120 ms). Precursors were fragmented by HCD using 32% normalized collision energy (NCE). Dynamic exclusion was performed for 45 s to limit repeated peptide sequence. The raw data were acquired by data-dependent acquisition (DDA) analysis using Xcalibur software (version 2.5; Thermo Fisher Scientific). The mass spectrometry proteomics data have been deposited to the ProteomeXchange Consortium via the PRIDE^22^ partner repository with the dataset identifier PXD026994.

### Data analysis

All of 24 MS/MS data was processed using Proteome Discoverer (PD) version 2.4 (Thermofisher Scientific, Waltham, MA, USA) based on SEQUEST-HT search engine. The acquired data was compared and searched against the UniProtKB mouse database (SwissProt/TrEMBL, October_2014, 78,939 entries). The search parameters were as follows: complete trypsin digestion; precursor mass tolerance of 10 ppm and MS/MS tolerance of 20 ppm; false discovery rate (FDR) for all peptide-spectrum matches (PSMs) under 1%, and performed by Percolator software package (version 3.05) using delta Cn (0.05), strict FDR (0.01), relaxed FDR (0.05), and PEP (0.05) settings; fixed modifications were TMT tag (N-term and Lys, + 229.163 Da) and carbamidomethylation (Cys, + 57.021 Da); and variable modifications were oxidation (Met, + 15.995 Da) and deamidation (Asn and Gln, + 0.984 Da). Peptide groups were assigned to logical protein groups based on parsimony principle. The most confident centroid within 20 ppm of the expected mass of the reporter ions was used. TMT signals also were corrected for isotope impurities (Lot number: QL226165A). The identified proteins with PSMs that contained all intensity values from all TMT channels were used to obtain peptide quantification value. Common contaminants, such as keratin, were filtered out. The identified protein and quantification values were exported in the .csv format for further analysis.

### TMT relative quantification, normalization, and statistical analysis

TMT relative quantification analysis was performed using two quantification values derived from reporter ion quantification and PSM quantification. First, raw protein abundances were calculated as the simple summation of its associated and used peptide group abundance after the Reporter Ions Quantifier node in the Proteome Discoverer version 2.4 classify peptide groups. We then used the reporter ion intensities as estimates for protein abundance. For normalization using total peptide abundance, the Reporter Ions Quantifier node normalize the peptide groups and protein abundances by dividing abundances with the normalization factor over all samples. The normalization factor is the factor of the sum of the sample and the maximum sum in all files. After normalized reporter ion abundances (total peptide abundance) were imported into Perseus software version 1.6.10.43^23^, log2 transformation was performed. In addition, proteins with missing channels were filtered out. Then, Student’s t-test was performed to do identify differentially expressed proteins (DEPs), filtered using the following criteria: *p*-value < 0.05 and 1.2-fold change cutoff.

Next, raw reporter ion abundance extracted from the Proteome Discoverer was imported into Perseus software for width adjustment normalization strategy. After proteins with missing values are removed, the reporter ion abundances were transformed to log2 values, and the raw abundance data were normalized via width adjustment (this scales all values to equalize the interquartile ranges) in Perseus software. Then, Student’s *t*-test was performed to do identify differentially expressed proteins (DEPs), filtered using the following criteria: p-value < 0.05 and 1.2-fold change cutoff.

Finally, the proteins were also quantified and normalized based on PSMs by using MSstatsTMT^24^. The analysis was performed using the MSstatsTMT 2.2.0 and modified annotation file (Supplementary Table S1) in R version 4.1.2 with Bioconductor 3.14. First of all, we extracted PSMs from the Proteome Discoverer and embedded with annotation file which includes TMT related parameters such as run, channel, technical replicate mixture, channel, condition, mixture, and bio-replicates. The embedded data was used as input data and applied proteinSummarization function for MSstats normalization (default setting). Whereas total peptide amount and width adjustment normalization methods applies normalization only to protein summaries, known as protein-level normalization, MSstatsTMT performed spectrum-level normalization, protein summarization, and protein-level normalization separately. Then the normalized protein summaries were used as input to linear mixed-effects models with empirical bayes moderation^24^. The result was confirmed with profile plot of individual proteins from each fraction with dataProcessPlotsTMT function. The pairwise comparison was performed with groupComparisonTMT function. The results were filtered the following criteria: p-value < 0.05 and 1.2-fold change cutoff. After selection of DEPs in all analysis, principle component analysis (PCA) and hierarchical clustering were performed using the Perseus software.

### Bioinformatics analysis

Most biological process, molecular function, and pathway analyses were performed using DAVID^25^ and STRING version 11^26^. The identified biological processes were clustered using the EnrichmentMap^27^ and AutoAnnotate^28^ application in Cytoscape tool (version 3.7.2). Proteins detected by both quantification methods were analyzed using Venny 2.1.0 (bioinfogp.cnb.csic.es/tools/venny). Protein–protein interactions (PPIs) and visualization of protein expression with p-values were mapped using Cytoscape version 3.7.2. The KEGG pathway^29^ and PPI analysis were combined with STRING version 11 with BottleNeck method in cytoHubba^30^.

### Data independent acquisition (DIA) analysis

The peptide samples were re-dissolved in 10 μL of solvent A (0.1% formic acid in HPLC-grade water) and spiked with the index Retention Time (iRT) Kit (Biognosys AG, Schlieren, Switzerland). For DIA analysis, 1 ug of each sample was analyzed using a Q-Exactive HF-X mass spectrometer (Thermo Fisher Scientific) coupled with an Ultimate 3000 UHPLC system (Thermo Fisher Scientific). The MS1 scan was performed with the following parameters: scan range (m/z) = 390–1010; resolution = 60,000; AGC target = 1e6; maximum injection time = 60 ms. The DIA-MS/MS scan was performed in the HCD mode with the following parameters: isolation window of 8 Da without m/z overlap, precursor range = 390–1010 m/z, resolution = 15,000 with maximum injection time of 20 ms, AGC target = 1e6; normalized collision energy = 30%. The other MS parameters as well as LC gradient conditions and LC column were the same as those in DDA. DIA files were processed with the direct DIA experimental analysis workflow in Spectronaut^31^ v17 (Biognosys AG, Schlieren, Switzerland) against the UniprotKB mouse protein sequence database (UP000000589, April_2022, 63,628 entries) using default setting. The precursors and proteins with at a q-value of 0.01 from the mProphet model was considered for the identification of precursor and protein^32^. Interference correction on corrections at MS1 and MS2 level was levels were enabled, removing fragments/isotopes from quantification based on the presence of interfering signals but keeping at least three for quantification. All abundances were calculated based on the area under the extracted ion chromatogram (XIC) of all assigned fragments that passed filtering. The quantification of DIA data was performed by summing MS2 peak areas per each peptide with Spectronaut. Protein abundances were inferred by summing all MS2 intensities of the peptide that identified in three fractions. After log2 transformations, the protein abundance data were normalized via quantile normalization in Perseus software. Then, Student’s t-test was performed to identify differentially expressed proteins (DEPs), filtered using the following criteria: p-value < 0.05 and 1.5-fold change cutoff.

### Western blot validation

Two DEPs, Syt7 (synaptotagmin VII), and Map2 (microtubule associated protein 2) were selected for validation which were included in functional and interactome analysis of *Ewsr1* KO and WT mice. Equal amounts of proteins (approximately 20 μg) from three mice per group were separated by 10% SDS-PAGE gel electrophoresis. The proteins were transferred to a polyvinylidene fluoride membrane using a Mini Trans-Blot (Bio-Rad) over 90 min. The membranes were blocked with 5% BSA in TBS-Tween 20 (0.1%) at room temperature for 1 h and then incubated overnight at 4 °C with primary anti-Map2 (Cell Signaling, #4542, 1:1000), anti-Syt7 (Santa Cruz, sc-293343, 1:1000), and anti-GAPDH antibodies (Santa Cruz, sc-32233, 1:1000). The membranes were washed three times with TBS–Tween-20 (0.1%), 10 min each time, and incubated with HRP-conjugated secondary horse anti-mouse antibody (Cell Signaling Technology, 7076, 1:10,000) at room temperature for 2 h. After washing three times with TBS–Tween 20 (0.1%), 10 min each time, the membranes were processed using ECL detection reagents (GenDEPOT, W3652) and the protein bands detected using LAS-3000 system (Fujifilm, Japan). The ImageJ tool was used to define and measure the abundance of blots.

## Results

**Figure 1:**
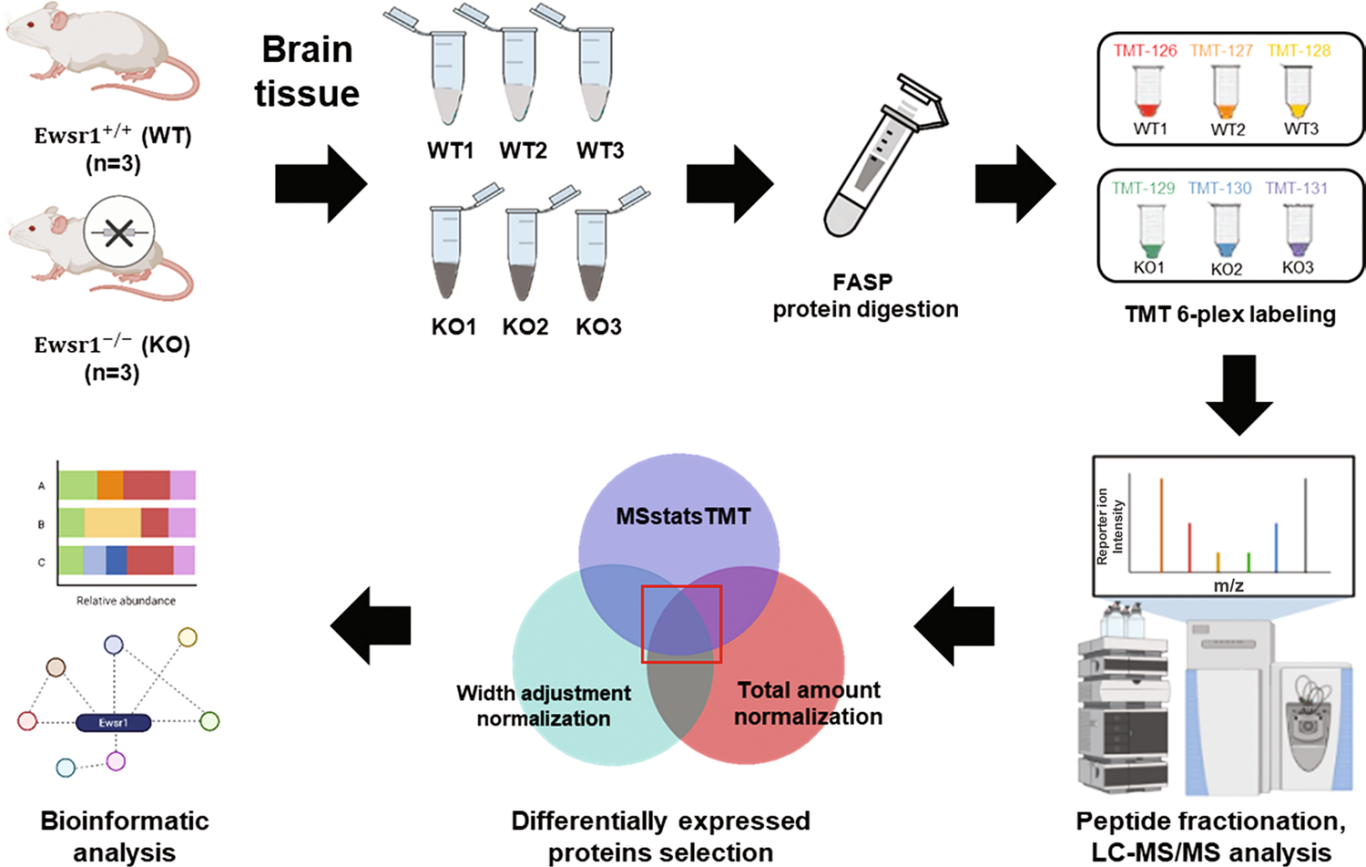
Overall proteome analysis scheme of the brain tissue from *Ewsr1* KO and WT mice. This figure was created with Biorender.com and exported under a paid subscription.

The aim of the current study was to gain insight into the molecular function and biological roles of EWSR1. Accordingly, we performed global proteome profiling of the brain tissue from *Ewsr1* KO and WT littermate control mice. Both *Ewsr1* KO and WT mice were generated and gene KO was validated by previous study describing genotyping and investigation of neuronal nucleus size of brain sections^11,10^. For quantitative proteomic analysis, we used 6-plex TMT isobaric labeling, with high-pH fractionation to increase protein identification efficiency. Protein samples of brain tissue from each mouse were individually labeled and analyzed by MS. The data were analyzed using protein sequence database search and various bioinformatics tools (Fig. 1).

### Quantitative proteome analysis of *Ewsr1* KO versus WT mouse brain tissue

**Figure 2:**
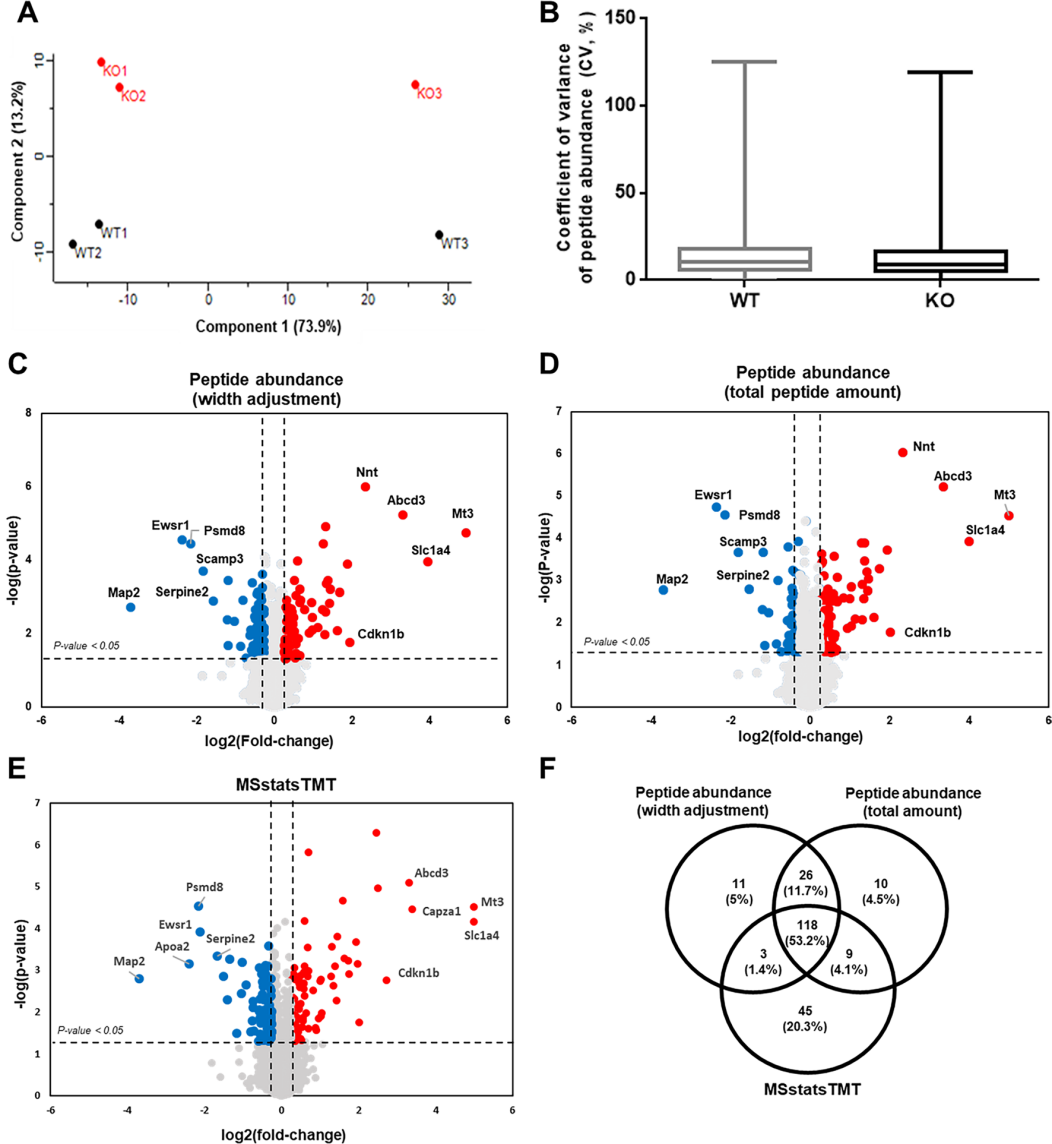
Quantitative proteomics analysis of Ewsr1 KO and WT mice. (**A**) Principle-component analysis (PCA) plot. (**B**) Coefficient variance (CV) plot from raw data of each individual MS analysis. Volcano plots for differential protein expression. Differential expression selected by peptide abundance comparison followed with (**C**) width adjustment normalization and (**D**) total peptide amount normalization. (**E**) Differential expression selected by MSstatsTMT. The x-axis of volcano plot indicates ${\mathrm{log}}_{2}(\mathrm{fold change})$ and two dashed vertical lines mark the 1.2-fold change cutoff and indicate statistical significance; − log_10_(*p*-value) and the dashed horizontal line equals to *p* = 0.05. (**F**) Common DEPs from measuring peptide abundance based on width adjustment and total adjustment normalization and monitoring each PSMs by MSstatsTMT.

We compared the global proteome of *Ewsr1* KO mouse brain tissue with that of WT mice using isobaric TMT labeling with LC–MS/MS. PCA of MS data showed good separation of *Ewsr1* KO and WT mouse brain samples **(**Fig. 2A). Overall, 9115 proteins were identified and 9025 proteins were quantified with the protein and peptide level FDR below 1% (FDR Q-value < 0.01) using the multiplexed TMT approach, with a stable coefficient of variance (CV) of raw peptide abundance during the analysis (Fig. 2B, Table S2).

To identify proteins that are potentially involved in the *Ewsr1* mediated cellular process, we used three different strategies, i.e., quantifying of peptide abundance based on intensities of the reporter tags using width adjustment or total amount normalization at protein-level, and quantifying of peptide abundance from each protein at PSM-level using normalization at spectrum- and protein-level by MSstatsTMT^24^ (Fig. S1). The box plots show that the three different methods perform as expected and make the centroids of the global distributions more similar (Fig. S2). In addition, we compared CV distributions for the original data, after width adjustment normalization, total amount normalization, or MSstatsTMT. In the original data, the averages CVs of the WT and KO groups were 14.19% and 15.02%, respectively (Fig. 2B), whereas the three normalization methods dramatically lower the average CVs, ranging from 0.92% to 6.9% (Fig. S3). Interestingly, the MSstatsTMT showed a very good behavior with a lowest CV value, whereas the width adjustment normalization displayed a greater dispersion of data compared with other methods (Fig. S3). However, all three methods performed well, showing relatively lower CV values.

During comparison, we selected proteins that were significantly up-regulated (p-value < 0.05 and fold-change > 1.2) or down-regulated (p-value < 0.05 and fold-change <  − 1.2). This revealed 158 (up = 73, down = 85) and 163 DEPs (up = 84, down = 79) identified by width adjustment normalization and total abundance normalization, respectively, and 175 DEPs (up = 80, down = 95) identified by MSstatsTMT analysis. All of the detected DEPs were visualized using volcano plots (Fig. 2C–E). All DEPs are listed in Tables S3–S5. Figure 2F also shows the number of differentially expressed proteins found using the same criteria for the data with different normalization procedures and common proteins from three different methods. There was a good overlap between the normalization procedures, and 118 DEPs were common to all the analyses. In other words, irrespective of normalization methods (with or without background normalization for TMT signal intensity, with or without quantile normalization of the summed protein intensity, and with or without spectrum-level normalization) these 118 proteins were differentially expressed in *Ewsr1* KO mouse brain compared to wildtype mice.

### Physiological process and molecular function of *Ewrs1* KO mouse brain proteome

**Figure 3:**
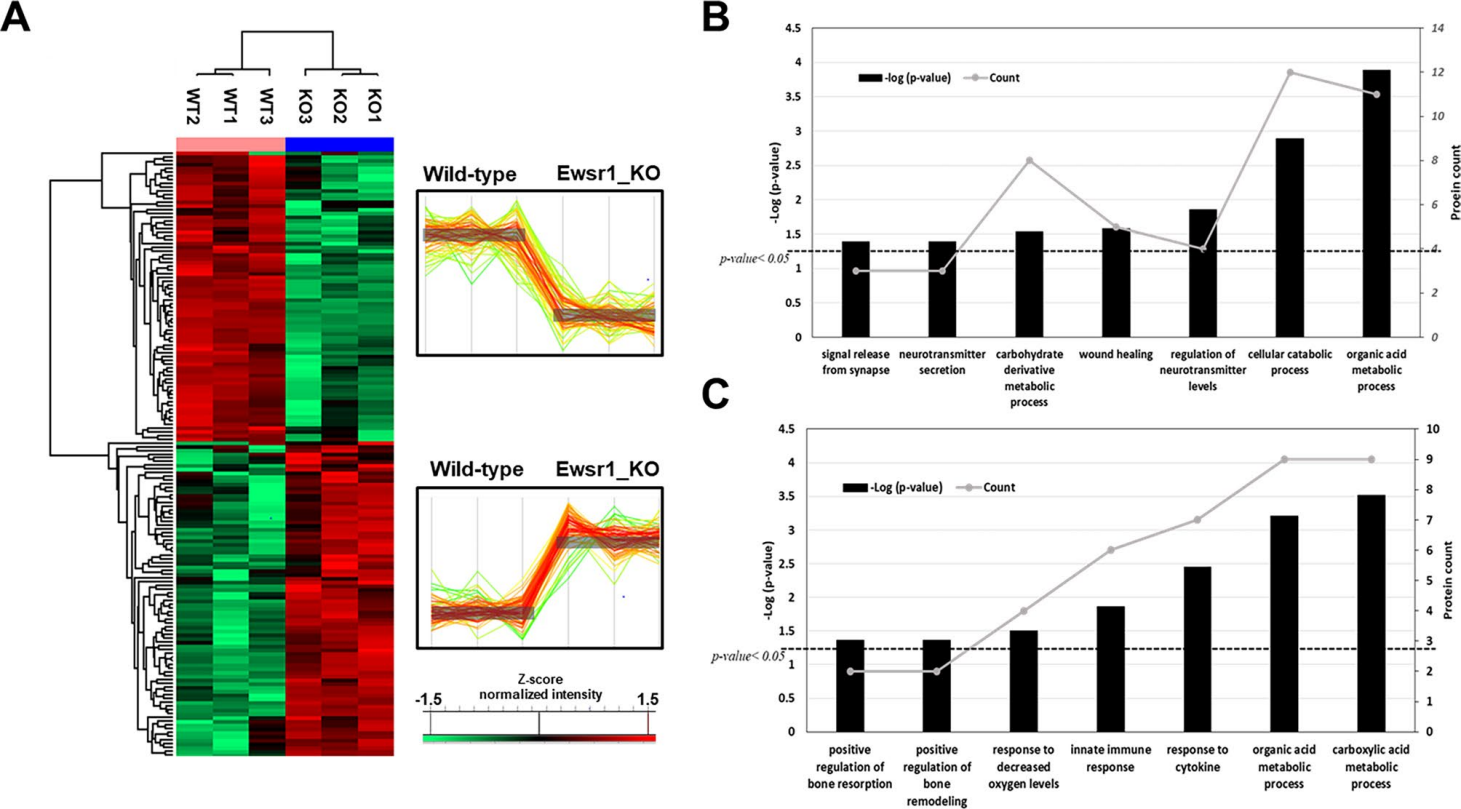
Hierarchical clustering of common DEPs in three different normalization methods. (**A**) Heatmap representation of the 118 common DEPs of *Ewsr1* WT and KO mice with colorimetric scheme. Each column represents mice sample and each row represents protein. Hierarchical clustering and heatmap generation were conducted using Perseus software. Hierarchical clustering was perform using the normalized protein intensities calculated from MSstatsTMT. Two major clusters were detected based on protein expression and overall expression aspects of those. The first cluster contains the proteins which were significantly down regulated with *Ewsr1* KO and second cluster contains significantly down regulated proteins by *Ewsr1* KO. DAVID analysis results of cluster 1 (**B**) and cluster 2 (**C**). The p-value was presented with the bar chart and protein counts were indicated with line charts. The dashed line in (**B,C**) represents p < 0.05.

To uncover the physiological or functional characteristics associated with specific changes in the *Ewsr1* KO mouse brain, we first concentrated on the 118 DEPs that were identified in all three statistical analyses. We generated a hierarchical clustering map of the 118 DEPs using normalized protein intensities that were calculated from MSstatsTMT and visualized the protein expression patterns in the brain tissue with Perseus software (version 1.6.10.43) (Fig. 3A). The hierarchical clustering of 118 DEPs using normalized protein intensities obtained from width adjustment and total peptide normalization is also shown in Fig. S4. Sample clusters by column were clearly separated into two large clusters, *Ewsr1* KO or WT, and protein expression patterns illustrated by the colorimetric scheme by row also indicated two major clusters. The first cluster contained proteins that were significantly down-regulated in *Ewsr1* KO mouse brain and the second cluster contained significantly up-regulated proteins in *Ewsr1* KO mouse brain compared to that of WT mice. DEPs from each cluster were subjected to functional classification analysis using the web-based DAVID annotation tool (Tables S6, S7). The first cluster proteins were mostly categorized into metabolism-related biological processes, such as organic acid, carboxylic acid, and cellular amino acid metabolic processes. The major pathways determined by the KEGG and Reactome pathway analysis also indicated metabolic pathways, metabolism of amino acids and derivatives, and ABC-family protein-mediated transport pathways. In addition, the modulation of synaptic transmission and neurotransmitter transport were attained (Fig. 3B). Proteins up-regulated in *Ewsr1* KO brain tissue were also related to carboxylic acid and organic acid metabolic processes. Specifically, positive regulation of bone resorption and remodeling functions were detected (Fig. 3C). This could be related to the physiological traits of EWSR1, a primary bone sarcoma that commonly occurs in the proximal long tubular bone or around the growth plate^33^.

**Figure 4:**
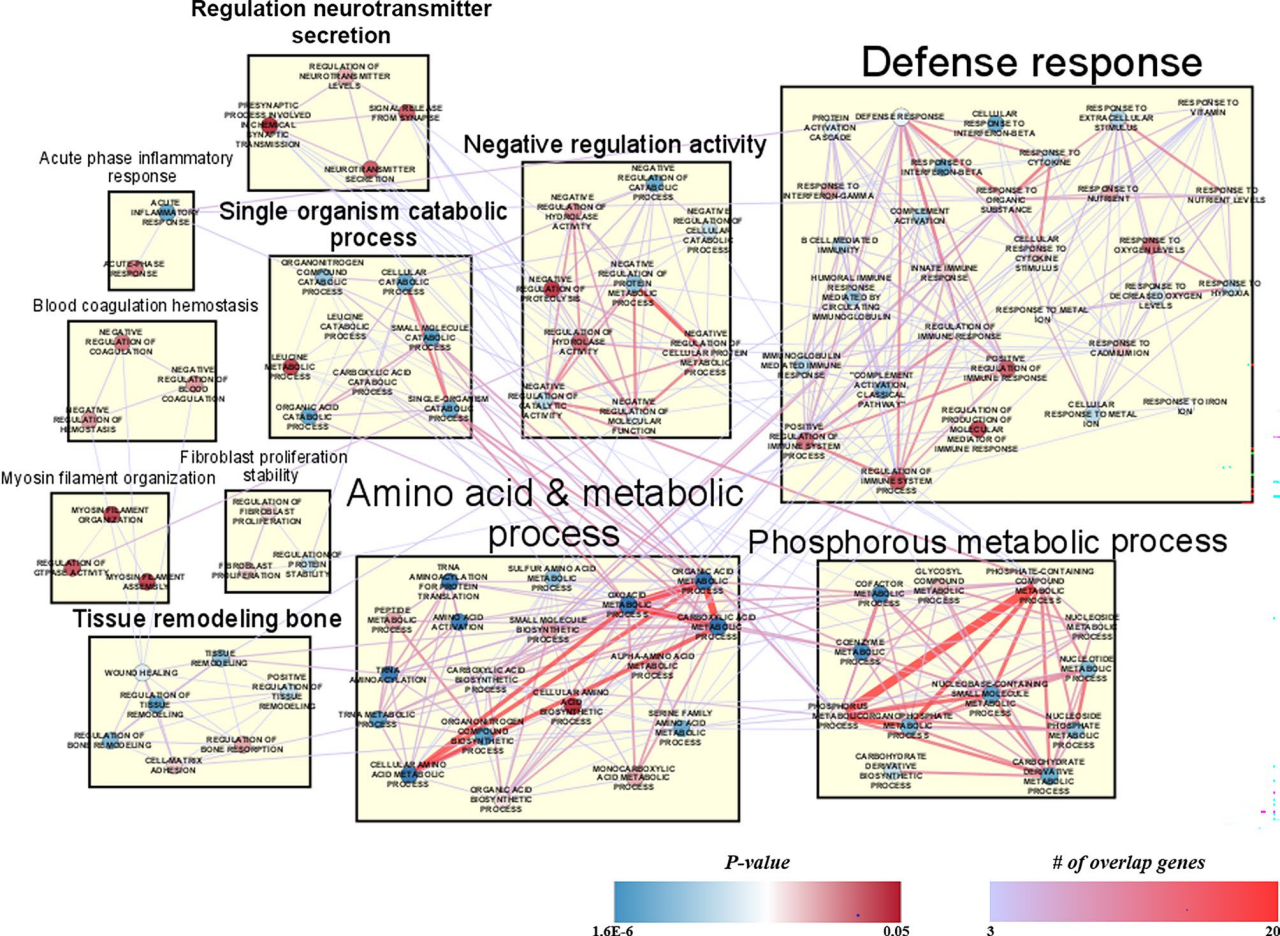
Enrichment map of DEPs with significant DAVID GO terms. EnrichmentMap was performed using statistically significant GO terms (p < 0.05). All of nodes describes biological process in GO term with colorimetric scheme of p-value. The color and width of edges represents the number of genes overlapping between different pathways.

**Figure 5:**
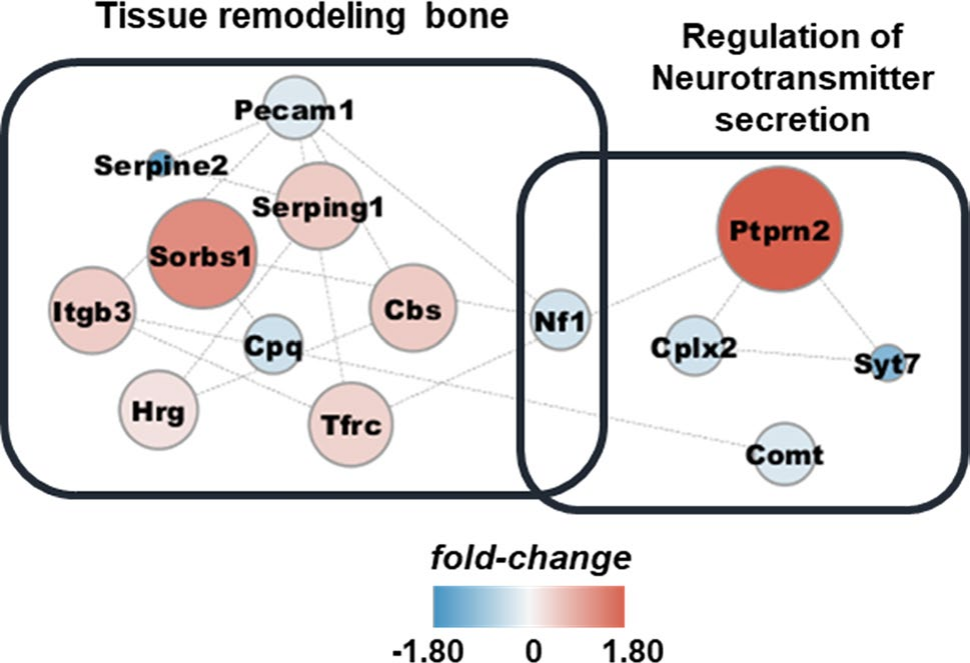
Protein–protein interaction of tissue remodeling bone and regulation of neurotransmitter secretion process related proteins. The colorimetric scheme and size of node reflects the protein expressions.

Furthermore, we used EnrichmentMap^27^ and gene ontology (GO) to analyze all DEPs to investigate functionally relevant proteins, coherence pathways, and network interactions by implementing statistically significant (p-value < 0.05) DAVID terms (Fig. 4). We visualized the protein network and integrated it within the Cytoscape tool^34^. The identified GO terms were categorized into 11 clusters. The majority of the enriched functions were correlated with metabolic pathways, such as the phosphorous metabolic pathway, which was the most statistically significant enriched term. This could be correlated with the results of a microRNA study, which showed that *Ewsr1* KO in the spinal cord leads to the deregulation of G-protein signaling and affects metabolic pathways^35^. Among these pathways, the regulation of neurotransmitter secretion and bone remodeling were enriched, which were correlated with the DAVID analysis outcomes. The neurotransmitter secretion cluster includes the regulation of presynaptic processes, signal release from synapses, and neurotransmitter level processes. By contrast, regulation of bone remodeling, cell–matrix adhesion, and wound healing functions were classified into tissue remodeling: bone clusters. Therefore, we concentrated on these two physiological processes and extracted the proteins allocated to these clusters to investigate their expression patterns. Five and ten proteins were classified into neurotransmitter secretion and tissue remodeling: bone processes (Table 1), respectively. The expression patterns and protein interactions were visualized using the Cytoscape tool (Fig. 5). Overall, proteins involved in the tissue remodeling: bone function were up-regulated and those involved in the regulation of neurotransmitter secretion process were down-regulated in the brain of *Ewsr1* KO mice.

**Table 1:** Biological process and gene categorized in tissue remodeling bone and neurotransmitter regulation process.

Enrichment	Term	Count	%	p-value	Genes	Fold enrichment	GO
Tissue remodeling bone	Wound healing	8	5.517	0.021	SERPINE2, CPQ, PECAM1, NF1, HRG, SERPING1, SYT7, ITGB3	2.869	GO: 0042060
	Positive regulation of tissue remodeling	3	2.069	0.017	TFRC, HRG, ITGB3	15.064	GO: 034105
	Regulation of bone remodeling	4	2.759	0.006	TFRC, NF1, SYT7, ITGB3	10.590	GO: 0046850
	Cell–matrix adhesion	5	3.448	0.032	SORBS1, PECAM1, NF1, HRG, ITGB3	4.161	GO: 0007160
	Regulation of bone resorption	3	2.069	0.036	TFRC, NF1, ITGB3	9.928	GO: 0045124
Regulation of neurotransmitter secretion	Signal release from synapse	4	2.759	0.044	CPLX2, PTPRN2, NF1, SYT7	5.065	GO: 0099643
	Neurotransmitter secretion	4	2.759	0.044	CPLX2, PTPRN2, NF1, SYT7	5.065	GO: 0007269
	Presynaptic process involved in chemical synaptic transmission	4	2.759	0.049	CPLX2, PTPRN2, NF1, SYT7	4.854	GO: 0099531
	Regulation of neurotransmitter levels	5	3.448	0.035	CPLX2, PTPRN2, NF1, COMT, SYT7	4.023	GO: 0001505

### Protein–protein interaction analysis of *Ewrs1* KO mouse brain proteome

**Figure 6:**
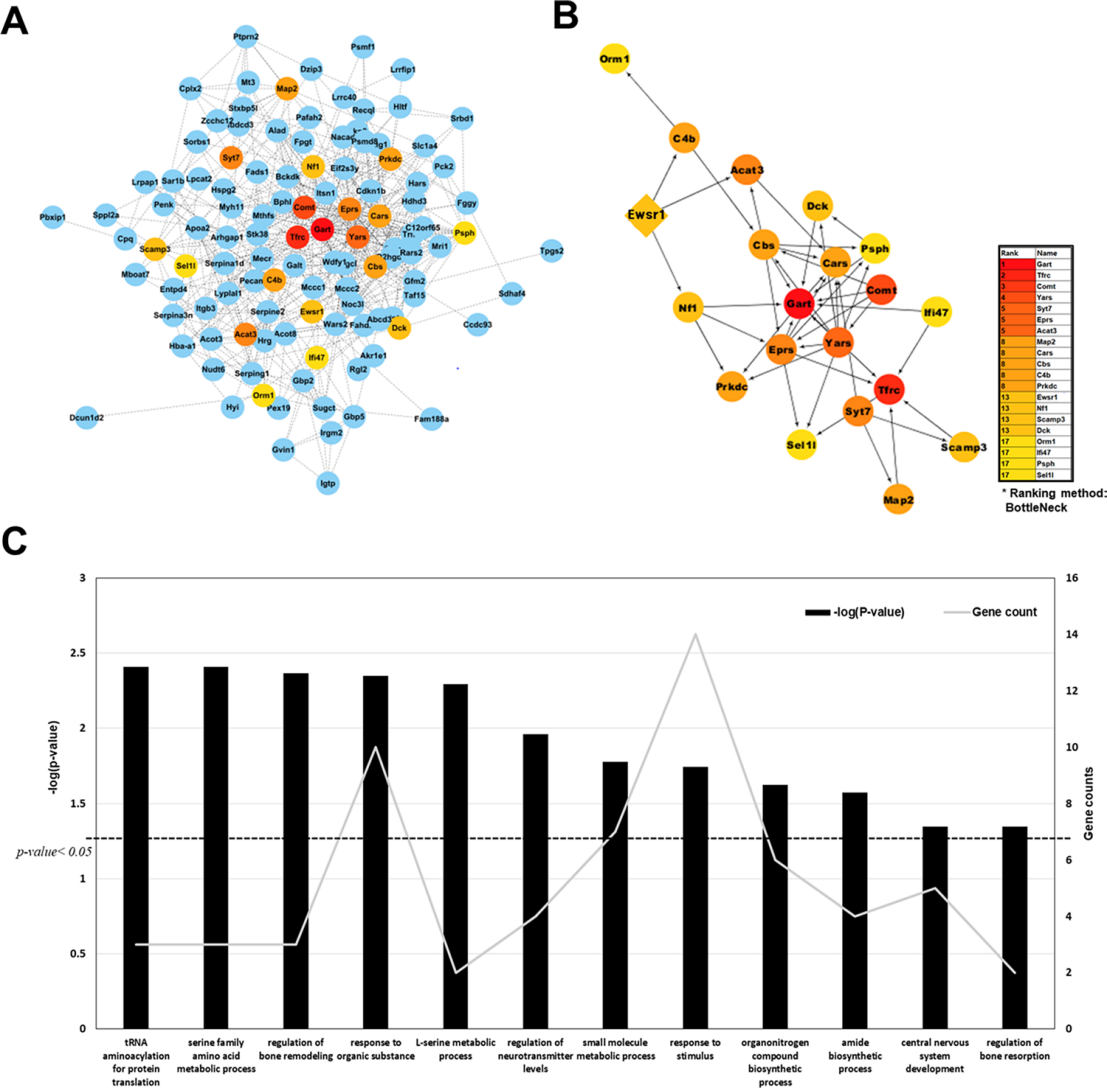
Interactome analysis of significantly altered proteins in *Ewsr1* KO mouse brain. (**A**) The expanded sub-network PPI of *Ewsr1* KO mouse brain marked with BottleNeck ranking method. (**B**) The extracted interactome of top 20 rank of BottleNeck method marked with colorimetric scheme. (**C**) DAVID functional analysis of top 20 interactome analysis. The p-value was presented with the bar chart and protein counts were indicated with line charts. The dashed line in (**C**) represents *p* < 0.05.

Although we have identified a few enriched biological processes for the differentially expressed proteins, we still could not rule out key biological process and molecules in *Ewsr1* KO mouse brain proteome. For this, we conducted an interactome analysis with 156 DEPs that identified in two statistical methods or more to unravel key functional modules using the STRING version 11^26^. We used the protein–protein interaction (PPI) with co-expression, co-occurrence, neighborhood, and embedded databases. The required PPI confidence was calculated using the BottleNeck ranking method, embedded in cytoHubba^30^ and top 20 hits with the shortest path between each protein were filtered using a colorimetric scheme. The layout was refined using Prefuse force-directed layout. The expanded sub-network of interacting proteins (Fig. 6A) and the top 20 proteins were plotted, showing that Ewsr1 directly interacted with Nrf1, C4b, and Acat3 (Fig. 6B). Interestingly, the GO enrichment showed that the Ewsr1 sub-network is associated with CNS development and regulation of bone resorption (Fig. 6C). Especially, Syt7 (synaptotagmin 7) and Nf1 (neurofibromin) showed a high rank of interaction correlation, and both proteins were downregulated in the brains of *Ewsr1* KO mice (Tables S3–S5).

### Validation of global proteome experiments

**Figure 7:**
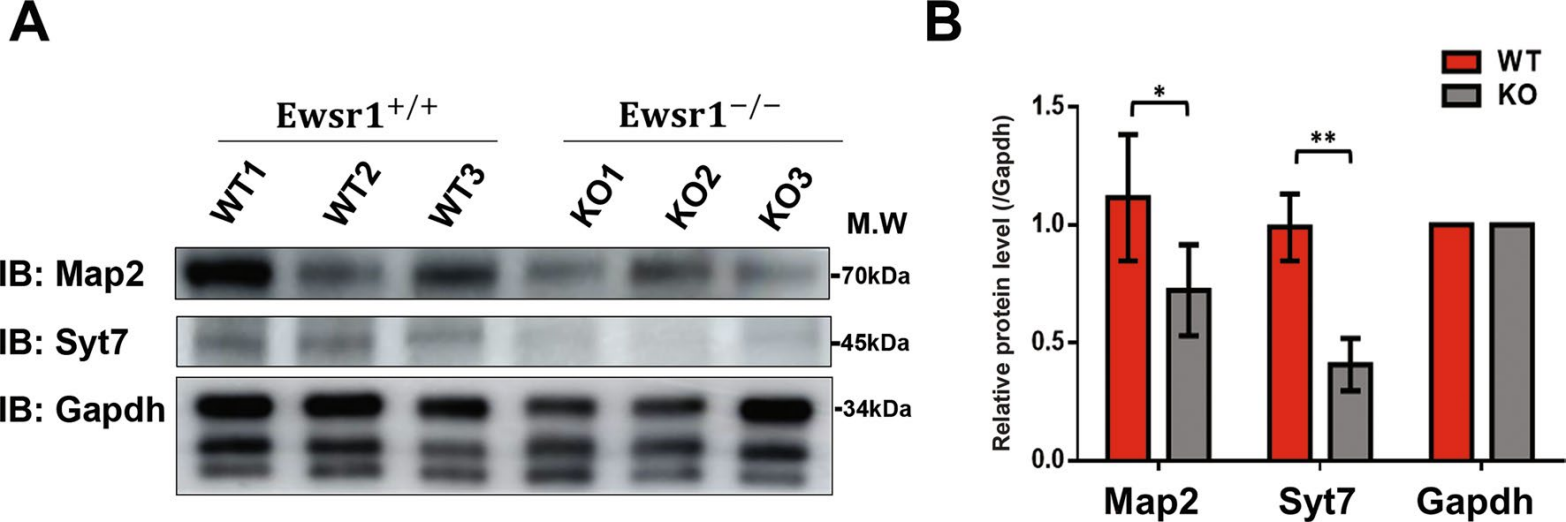
Validation of proteomics results. (**A**) Western blot validation of Map2 and Syt7. The result from LC–MS/MS and interactome analysis were validated by western blot assay. We selected Syt7 which was one of the most down-regulated protein by *Ewsr1* KO and included in top 20 interacted proteins with Ewsr1. Map2 was also highly down-regulated proteins compared with other DEPs by *Ewsr1* KO. Gapdh was used as the loading control. (**B**) Bar graphs showing normalized protein levels of validated targets. Red color represents wild type (WT) mouse brain tissues and black color represents EWSR1 knock-out (KO) brain tissues. Results are plotted as means ± standard deviations of values from three independent experiments. Student’s *t*-test was used for statistical analysis (*p < 0.05, and **p < 0.005).

The GO analysis and interactome analysis revealed that the key biological processes, tissue remodeling and regulation of neurotransmitter process could be affected by *Ewsr1*. Moreover, we found highly interacting proteins were predicted to directly interact with *Ewsr1*. Therefore, we reasoned that if we could reveal identical expression alterations from an independent proteomic analysis, we could have much more confidence in our results. Data-independent acquisition (DIA)-MS was used for orthogonal validation of our finding from TMT quantification^36^. DIA-MS analysis was performed in the independent samples consisting of three wild-type and three knock-out mice. Evaluation of reproducibility and quantitative ability of our DIA analysis indicated high reproducibility (Fig. S5A,B). Significantly differentially regulated targets in *Ewsr1* KO relative to WT showed good agreement between TMT and DIA datasets (Fig. S5C, Table S7). Interestingly, nine out of 20 top interacting proteins highlighted in Fig. 6B were observed as DEPs in DIA-MS experiments, indicating the reliability of our analysis (Fig. S5D). In addition, two proteins (Map2 and Syt7) were validated by western blotting We selected Map2 as proteins that were highly downregulated in Ewsr1 KO mouse brain compared with other DEPs (Tables S3, S4). Syt7 was selected because it is involved in both terms of tissue remodeling bone and regulation of neurotransmitter secretion. Furthermore, these two proteins were predicted to have confidentially interaction with *Ewsr1*. The analysis revealed that all these proteins were down-regulated in the brain of *Ewsr1* KO mice (Fig. 7, Fig. S6). The *t*-test results and abundance of each blotted band are presented in Table S9. These replicated experiments implied that the Ewsr1 and its interacting proteins discovered in the TMT-labeling experiment are crucial with high confidence.

## Discussion

In the current study, we elucidated the role of Ewsr1 in the mouse brain by comparing *Ewsr1* KO and WT mouse brains using proteomics. To increase the confidence of the obtained results, we used three different methods to identify DEPs in *Ewsr1* KO and WT mouse brains. We employed those normalization approaches to maximize the identification of representative proteins and for cross-validation of proteome data. In proteomic experiments, TMT-labeled analysis may involve numerous biological replicates that cannot be handled in a single run, multiple TMT mixtures or analyses. Such large-scale analysis can lead to apparent biological and technical variations in the TMT-based workflow and affect downstream statistical processes. Therefore, to eliminate biological or technical variances during analysis, we first conducted width adjustment normalization, which calculates the quantiles (q1, q2, and q3) from all the values, and set the median (q2) as a standard to rearrange the distributions. Next, peptide abundance is normalized to the total sum of all spectrum intensities on the original scale for each protein and each channel^37^. The normalization factor is the sum of the sample and the maximum value in all raw files and equalizes the sums over all channels and analyses^38^. The total amount normalization calculates the scaling factors based on the total peptide ion abundance, and width adjustment normalization transforms the abundance of each ion to follow a data-derived reference distribution^39^.

MSstatsTMT was devised by Ting et al.^24^. It is a novel differential analysis design with spectrum-level normalization, unlike protein-level normalization, which is a conventional workflow for differential analysis of MS data. Moreover, MSstatsTMT analysis applies a linear mixed-effect model which is applicable for analysis with multiple variance components to develop stable conditions during analysis (Fig. S2). We used this method and compared it with the traditional statistical methods, i.e., proteomic analysis to identify the most representative changes in protein expression in *Ewsr1* KO mice (compared with WT). Consequently, we identified 158, 163, and 175 DEPs after width adjustment normalization, total abundance-based normalization in PD 2.2, and MSstatsTMT, respectively. Approximately 64.9% (144) of DEPs were identified by width adjustment and total abundance-based normalization, and 53.2% (118) of DEPs were commonly identified by the three methods. These results showed that different normalization approaches can significantly change the p-values and fold changes of a few proteins. Because all available approaches in this respect have pros and cons and none of them appeared to perform optimally in all situations, it is very importance to check every individual step of protein expression data analysis through comparison between different statistical analyses. Hence, we applied different normalization methods to detect the truly differentially expressed proteins in *Ewrs1* KO mouse brain, and as a result, commonly found proteins in three methods were selected for further analysis.

Next, we applied DAVID functional analysis to identify biological process changes by up and down-regulated proteins. The redundant biological process was removed and clustered with EnrichmentMap software to improve the interpretation of enrichment results. Most of the up-regulated proteins were involved in bone remodeling and positive regulation of bone resorption. Overexpression of one such protein, CDKN1b, in adult mouse using a doxycycline-inducible construct alters the balance between genome and tissue aging, and reduces cell proliferation in various tissues^40^. Another protein, integrin beta 3 (ITGB3) controls cellular senescence by promoting the TGF-β pathway, and its levels increase during aging in a subset of tissues^41^. By contrast, down-regulated proteins were correlated with the regulation of neurotransmitter secretion and presynaptic processes involved in chemical synaptic transmission. Map2 was the most highly down-regulated protein in the brain of *Ewsr1* KO mice. Interestingly, the prion disease leads to cell death mediated by the alteration of the MAP2/TAU family protein; in particular, the loss of Map2 is observed in prion disease and neuronal death, followed by microtubule disruption^42^. Another down-regulated protein, complexin 2 (CPLX2), is highly expressed in the CNS It also regulates synaptic neurotransmitter release, synaptic signaling^43^, plasticity^44^, and neuronal network functions^45^. Specifically, three *CPLX2* SNPs are associated with increased neural activity in the prefrontal cortex in patients with schizophrenia compared with healthy controls^46^. These observations either directly or indirectly support the results of the current study.

Furthermore, we investigated PPIs of Ewsr1 in the brain of *Ewsr1* KO mouse. We used the BottleNeck method embedded in CytoHubba to investigate the Ewsr1 interactome. The method assumes that the most frequently connected proteins channel the most critical information flow in the entire network^47^. Because the high score or rank lies on the number of shortest paths connecting different nodes in the network which suggest the protein may play a key role in mediating interactions between different protein complexes, we chose the top 20 proteins ranked by the BottleNeck method and performed their functional analysis. In proteins composed of the sub-network of Ewsr1, we observed that Syt7 (synaptotagmin 7) and Nf1 (neurofibromin) are downregulated in *Ewsr1* KO mouse brain. Asynchronous release of neurotransmitters is significantly slower in *Syt7* KO mouse than in WT mouse, without a specific difference in the total synaptic charge^48^. Further, the NF1 protein is found in various brain regions and plays a key role in neurofibromatosis. It also balances the formation of dendritic spines and is ubiquitously regulated by bone metabolism and tumorigenesis^49^.

It has been reported that TATA box-binding protein-associated factor (Taf15) co-localizes with Ewsr1 and leads to mitochondrial abnormalities, which we described in functional analysis section, and disrupts the neuromuscular junction^50^. *TAF15* is also a major gene for amyotrophic lateral sclerosis, which is a common neurodegenerative disease^51^. Although Taf15 did not reach the top 20 confidence rank, Ewsr1 directly interacted with Taf15. In addition, we observed that Taf15 protein was up-regulated in *Ewsr1* KO mouse brain (Fig. 6A). Furthermore, cyclin-dependent kinase inhibitor 1 B (CDKN1b) showed a relatively high connectivity and was up-regulated in *Ewsr1* KO mouse brain. Overexpression of *Cdkn1b* in adult mice reduces cell proliferation in multiple tissues, and also reduces the proliferation rate of neural stem cells and progenitor cells^52^.

Consequently, our result improves the understanding of Ewsr1-regulated interactions or biological processes in the brain regions and neurodegenerative diseases at the proteome level. However, to gain a deeper understanding of biological roles, it is also necessary to examine its transcriptomic^53^ and epigenomic^54^ profiles. The integration of the proteomics data with other high-throughput omics data can provide insights of the biological functions and pathology of *Ewsr1*. As Ewsr1 is well-known with major function in transcription and RNA splicing, RNA-seq analyses will help to elucidate gene regulatory mechanisms mediated by Ewsr1. Future investigations will be needed to demonstrate the critical roles of Ewsr1 at transcriptome and epigenome level. Finally, we expect that improved understanding of Ewsr1 and its protein interactions will advance the field of clinical research on neuronal development and differentiation.

### Supplementary Information

Supplementary Tables.

Supplementary Figures.

## Data Availability

All data generated or analyzed during the current study are available in the ProteomeXchange Consortium via the PRIDE partner repository (https://www.ebi.ac.uk/pride/archive/projects/PXD026994).
