# Supplementary material for: In-depth proteome analysis of brain tissue from Ewsr1 knockout mouse by multiplexed isobaric tandem mass tag labeling
Source: Sci Rep. 2023 Sep 14;13:15261. doi: 10.1038/s41598-023-42161-7 (PMC10502055; doi:10.1038/s41598-023-42161-7)
Supplement: Supplementary file 2 — Supplementary Figures. [file 41598_2023_42161_MOESM2_ESM.pdf]

Supplementary Figures

In-depth proteome analysis of brain tissue from *Ewsr1* knockout mouse by multiplexed isobaric tandem mass tag labeling

Jin Woo Jung[1,2,†], Hyeyoon Kim[1,2,†], Joonho Park[3], Jongmin Woo[4], Eunji Jeon[1,2], Geeeun Lee[2,5], Minseo Park[2],

Sarang Kim[2,5], Ho Seok Seo[2,6],Seongmin Cheon[1], Kisoon Dan[1], Junghee Lee[7], Hoon Ryu[7,8,], and Dohyun Han[1,2,9,*]*

[1] Proteomics core facility, Biomedical Research Institute, Seoul National University Hospital, Seoul, 03082, Korea

[2] Transdisplinary Department of Medicine & Advanced Technology, Seoul National University Hospital, Seoul, 03082, Korea

[3] Department of Pharmacology, CHA university College of medicine, Pocheon-si,11160, Korea

[4] Center for Translational Biomedical Research, North Carolina Research Campus, University of North Carolina at Greensboro, Kannapolis, NC, 28081, USA

[5] Department of Biomedical Sciences, Seoul National University Graduate School, Seoul, 03082, Korea

[6] Interdisciplinary Program in Neuroscience, College of Natural Sciences, Seoul National University, Seoul, 08826, Korea

[7] Boston University Alzheimer's Disease Center and Department of Neurology, Boston University School of Medicine, Boston, MA,02118, USA

[8] Brain Science Institute, Korea Institute of Science and Technology (KIST), Seoul, 02792, Korea

[9] Department of Medicine, College of Medicine, Seoul National University, Seoul, 03082, Korea

[†]J.W.J. and H.K. contributed equally to this work as co-first authors.

[*]H.R. and D.H. contributed equally to this work as co-corresponding authors.

Table of Contents

Page S-2: Supplementary Figure S1. Flow chart of data normalization and the process of differentially expressed proteins identification.

Page S-3: Supplementary Figure S2. Box plot comparison of $\log_2$ peptide abundance.

Page S-4: Supplementary Figure S3. Coefficient variation distribution of Width adjustment normalization, Total amount normalization, and MSstatsTMT normalization.

Page S-5: Supplementary Figure S4. Hierarchical clustering of differentially expressed proteins.

Page S-6: Supplementary Figure S5. Data-independent acquisition (DIA) proteomics.

Page S-7: Supplementary Figure S6. Densitometry plots for the western-blot analysis.

Page S-8: Supplementary Figure S7. Raw data of western blot analysis.

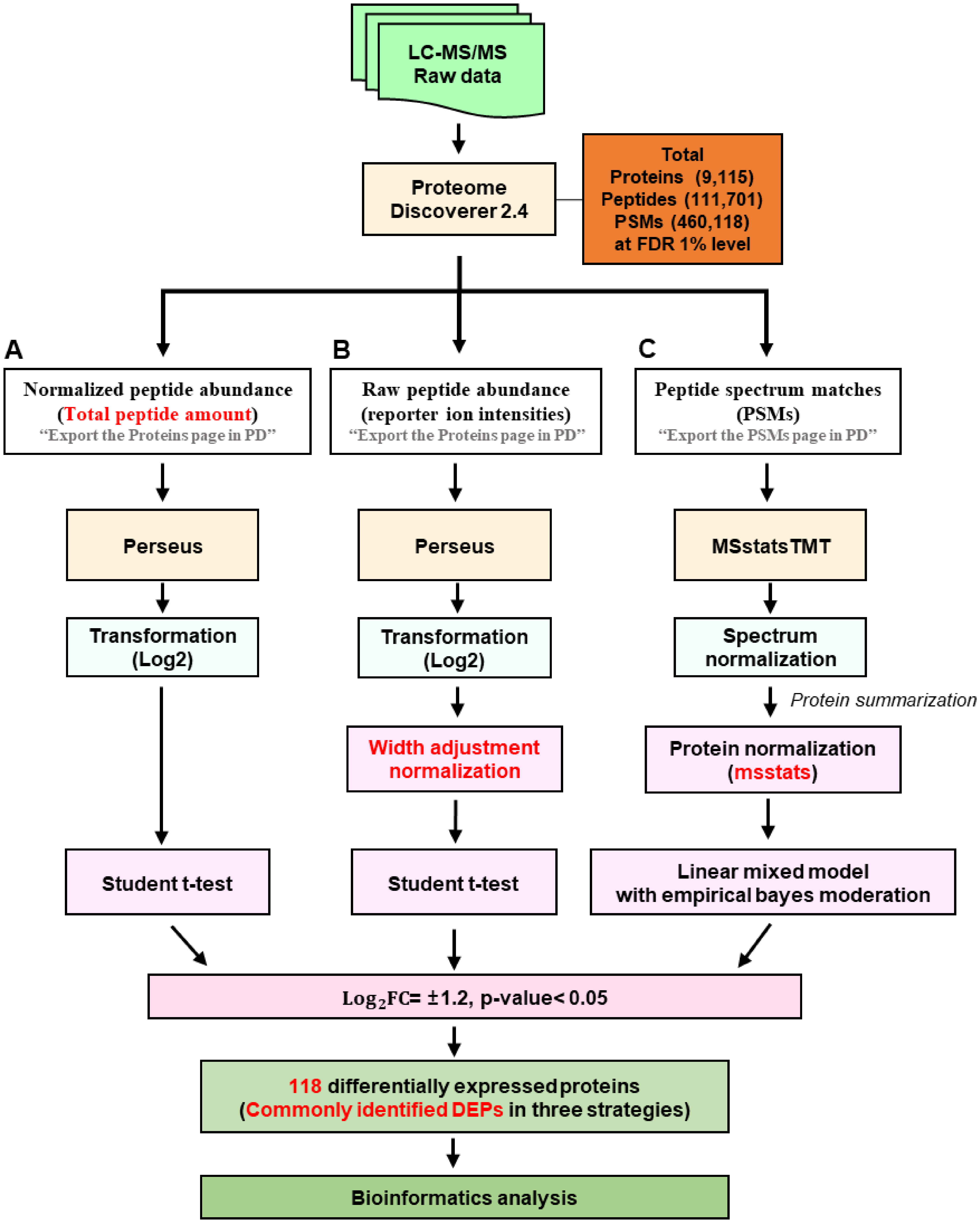

Supplementary Figure S1. Flow chart of data normalization and the process of differentially expressed proteins identification. During the identification of differentially expressed proteins, we applied two different quantification methods which use peptide abundance and peptide spectrum matches (PSMs). After logarithmic transformation, the Proteome Discoverer used adjustment and total sum of peptide abundance normalization to complement the variance. The MSstastsTMT used median normalization for stable comparison.

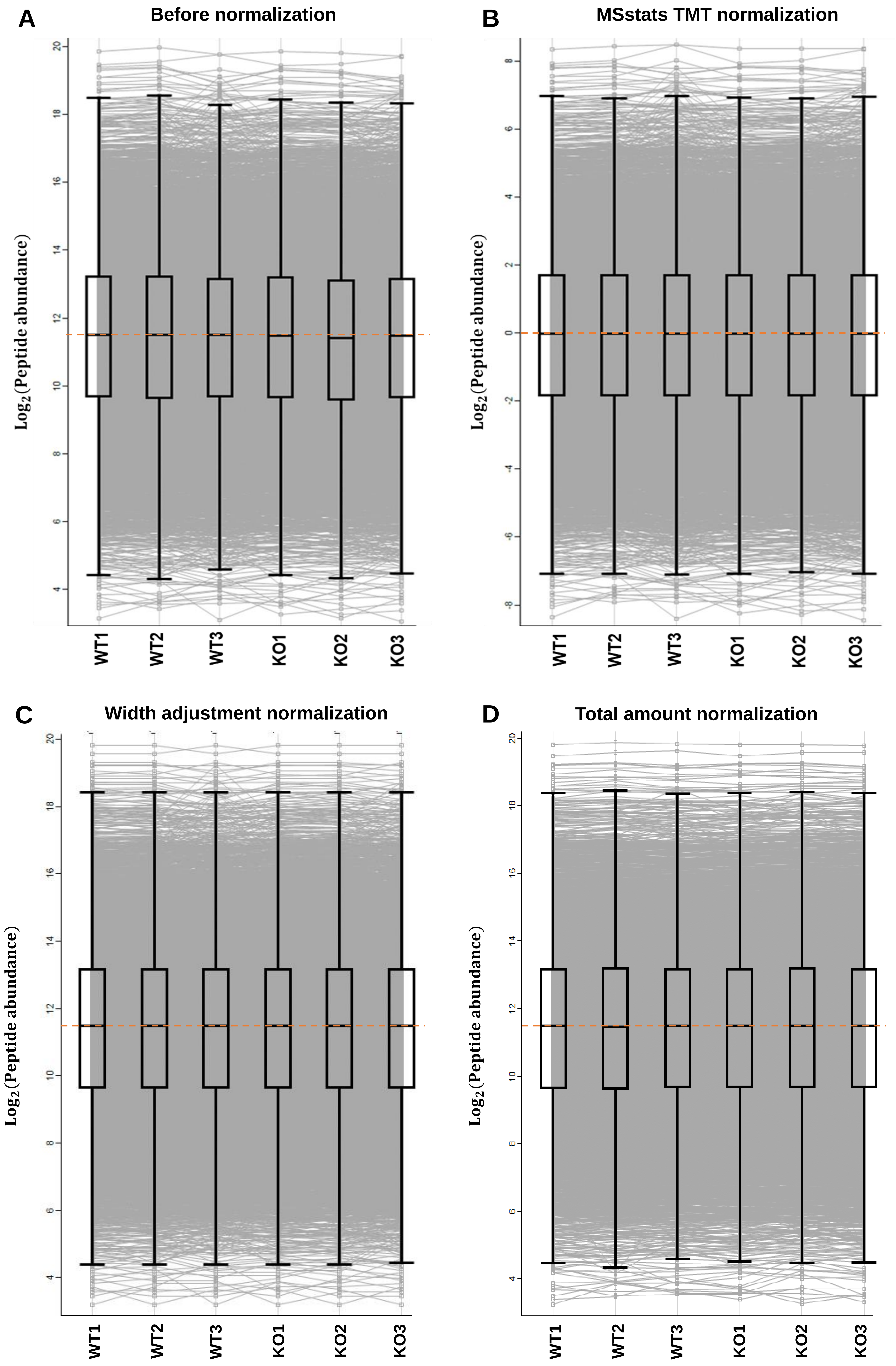

Supplementary Figure S2. Box plot comparison of log2 peptide abundance A) before normalization, after B) MSstatsTMT normalization, C) width adjustment normalization, and D) total amount normalization

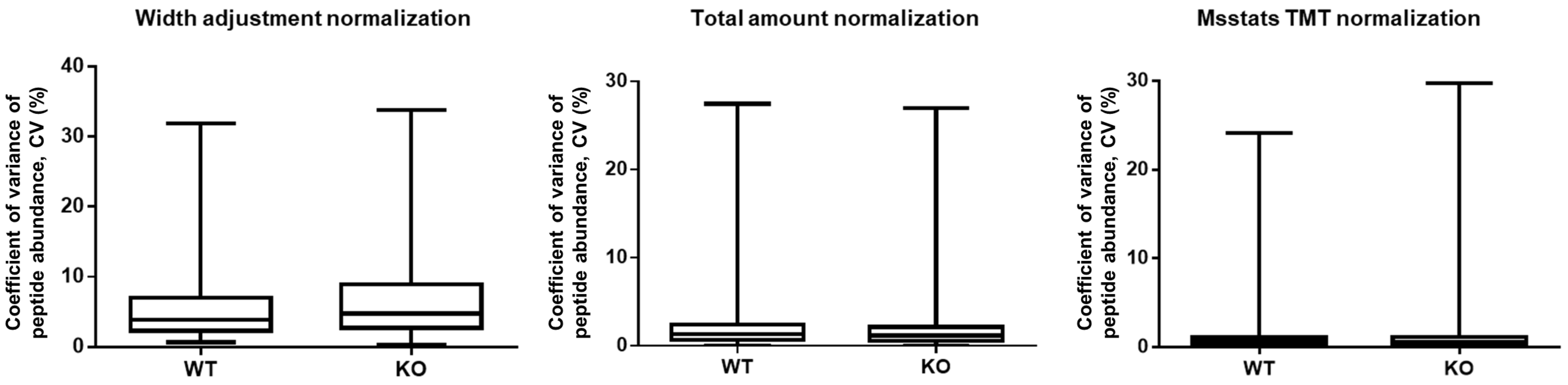

	Peptide abundance				PSMs	
	Width adjustment normalization		Total amount normalization		MSstatsTMT normalization	
**Sample group**	WT	KO	WT	KO	WT	KO
**Average CV (%)**	5.8584	6.8972	1.9031	1.6892	0.9570	0.9168

Supplementary Figure S3. Coefficient variation (CV) distribution of Width adjustment normalization, Total amount normalization, and MSstatsTMT normalization.

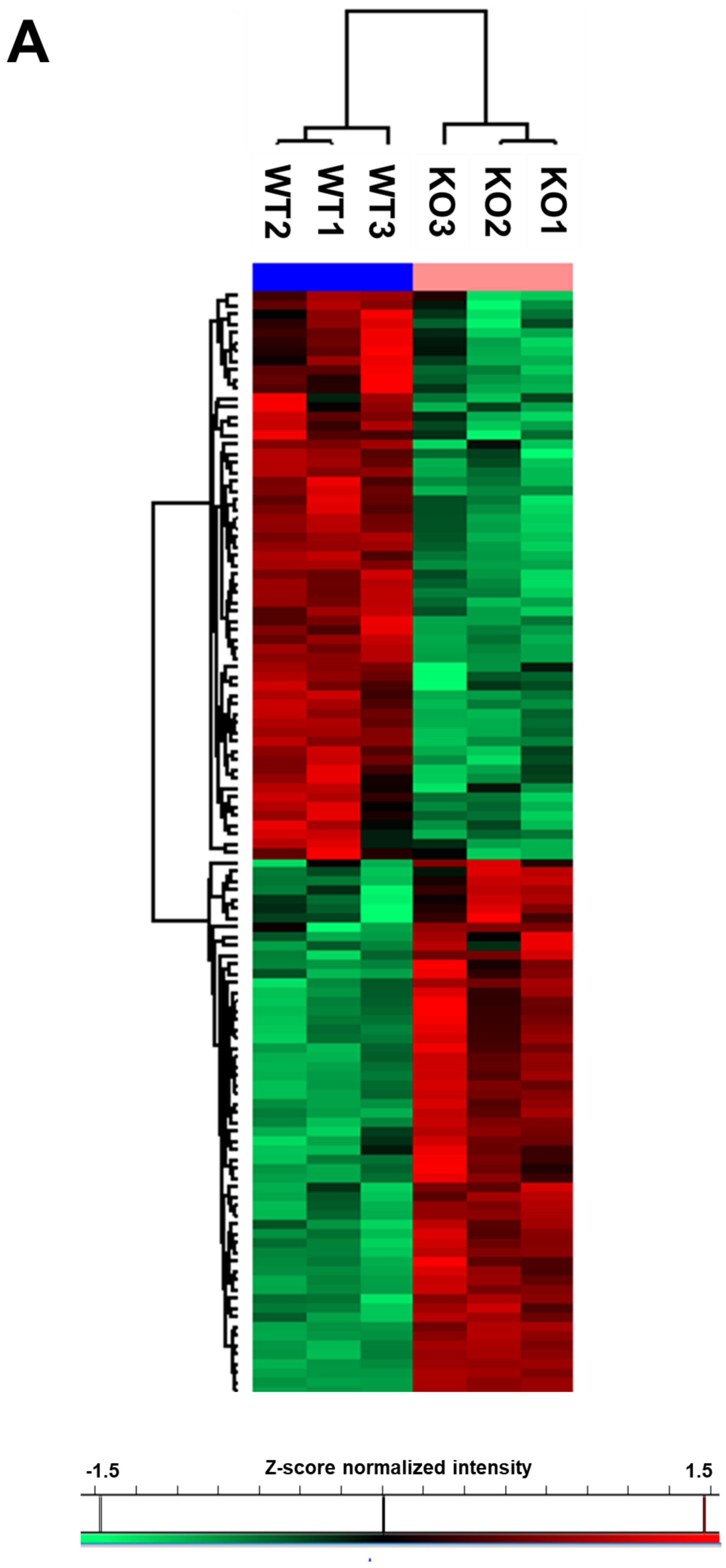

Supplementary Figure S4. Hierarchical clustering of differentially expressed proteins. A) Heatmap representation of protein expressions of *Ewsr1* WT and KO mice with colorimetric scheme from the width adjustment normalization. B) Heatmap representation of protein expressions of *Ewsr1* WT and KO mice with colorimetric scheme from total peptide abundance normalization. Hierarchical clustering and heatmap generation were conducted using Perseus software version 1.6.2.0 (https://maxquant.net/perseus/).

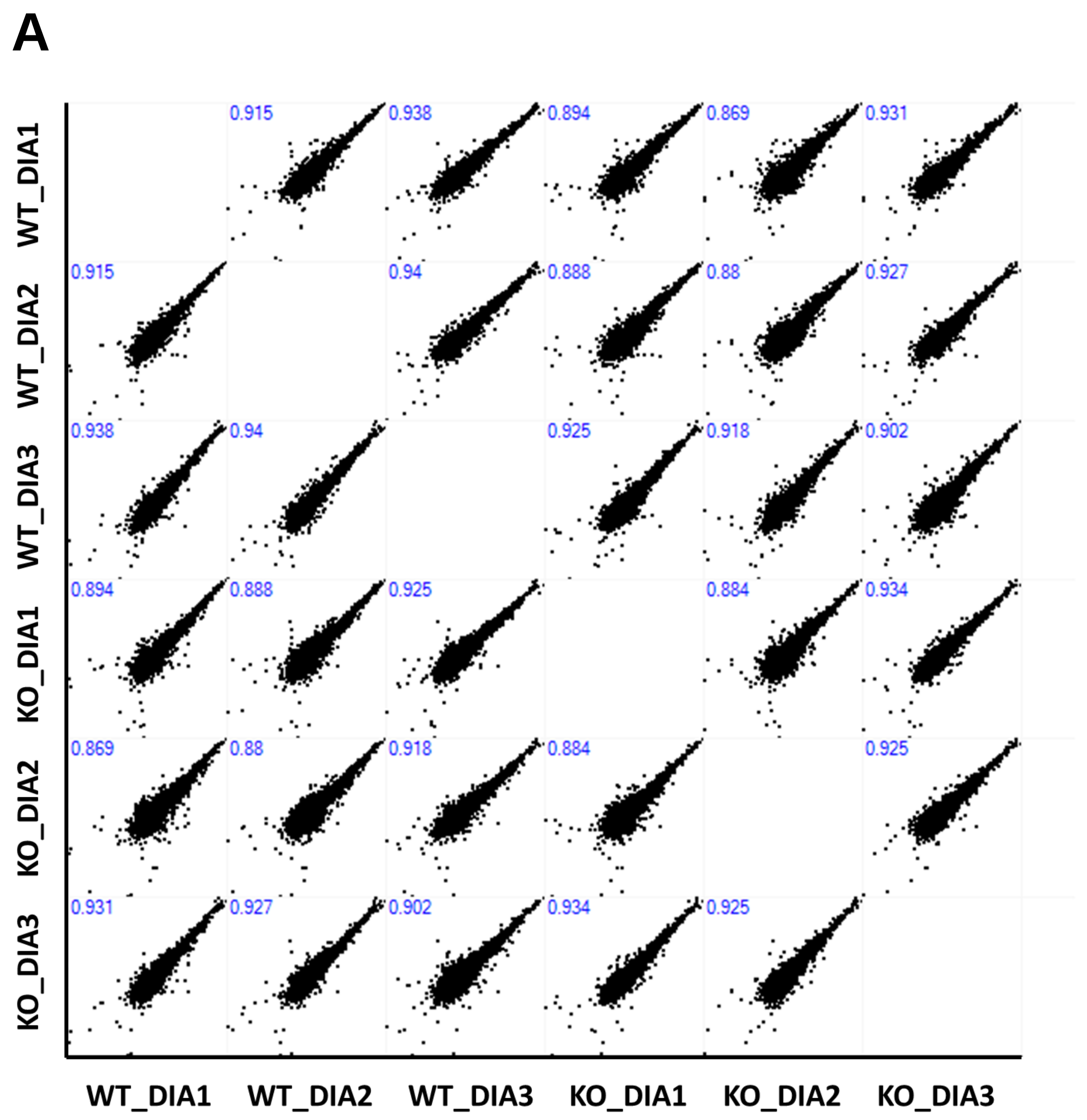

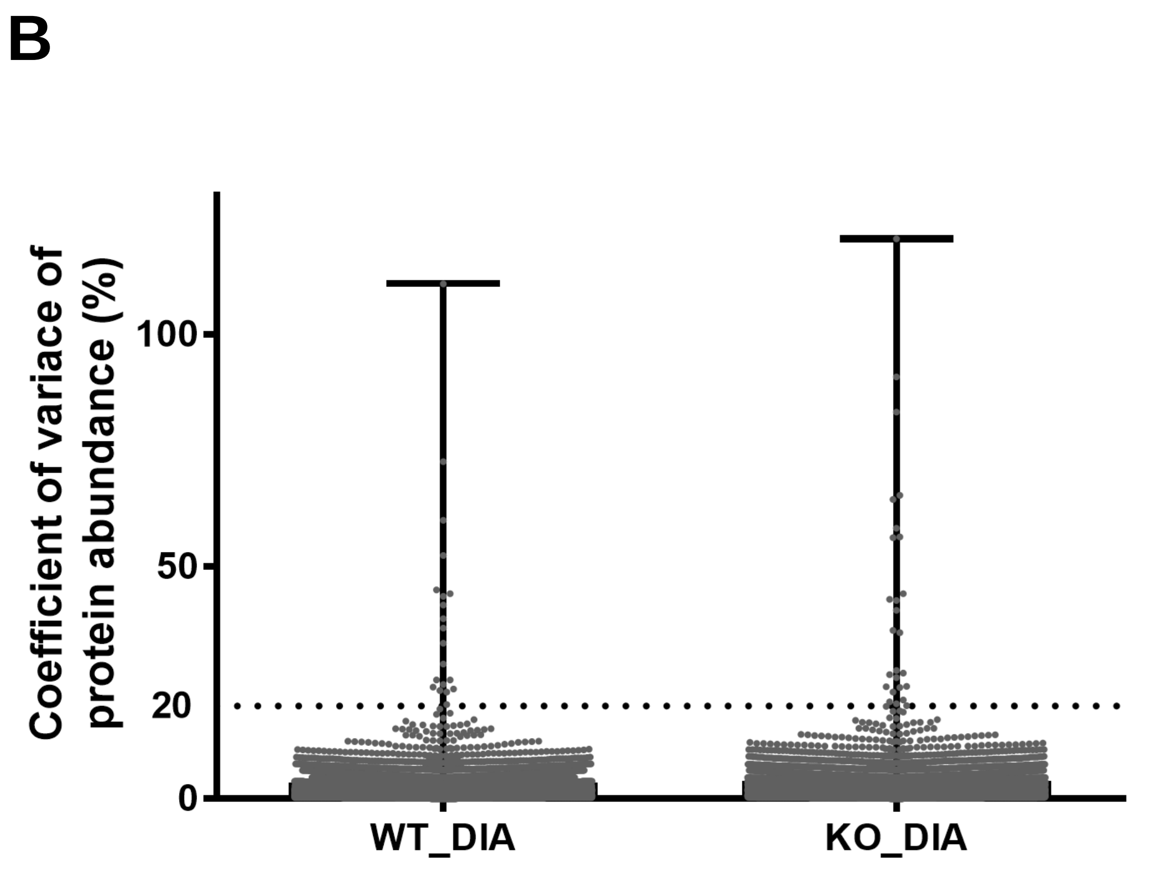

Sample group	WT	KO
Average CV (%)	2.268	2.535

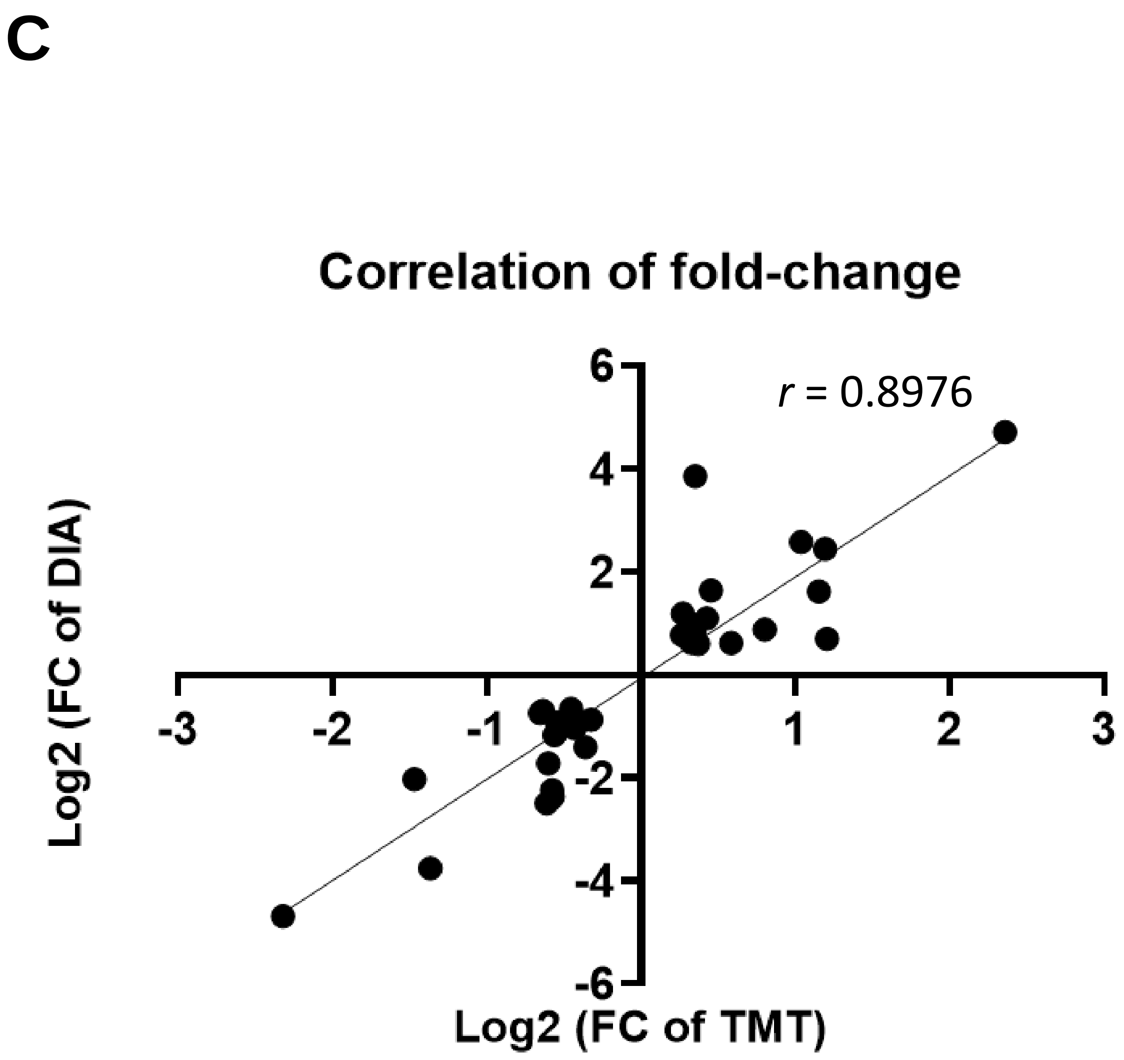

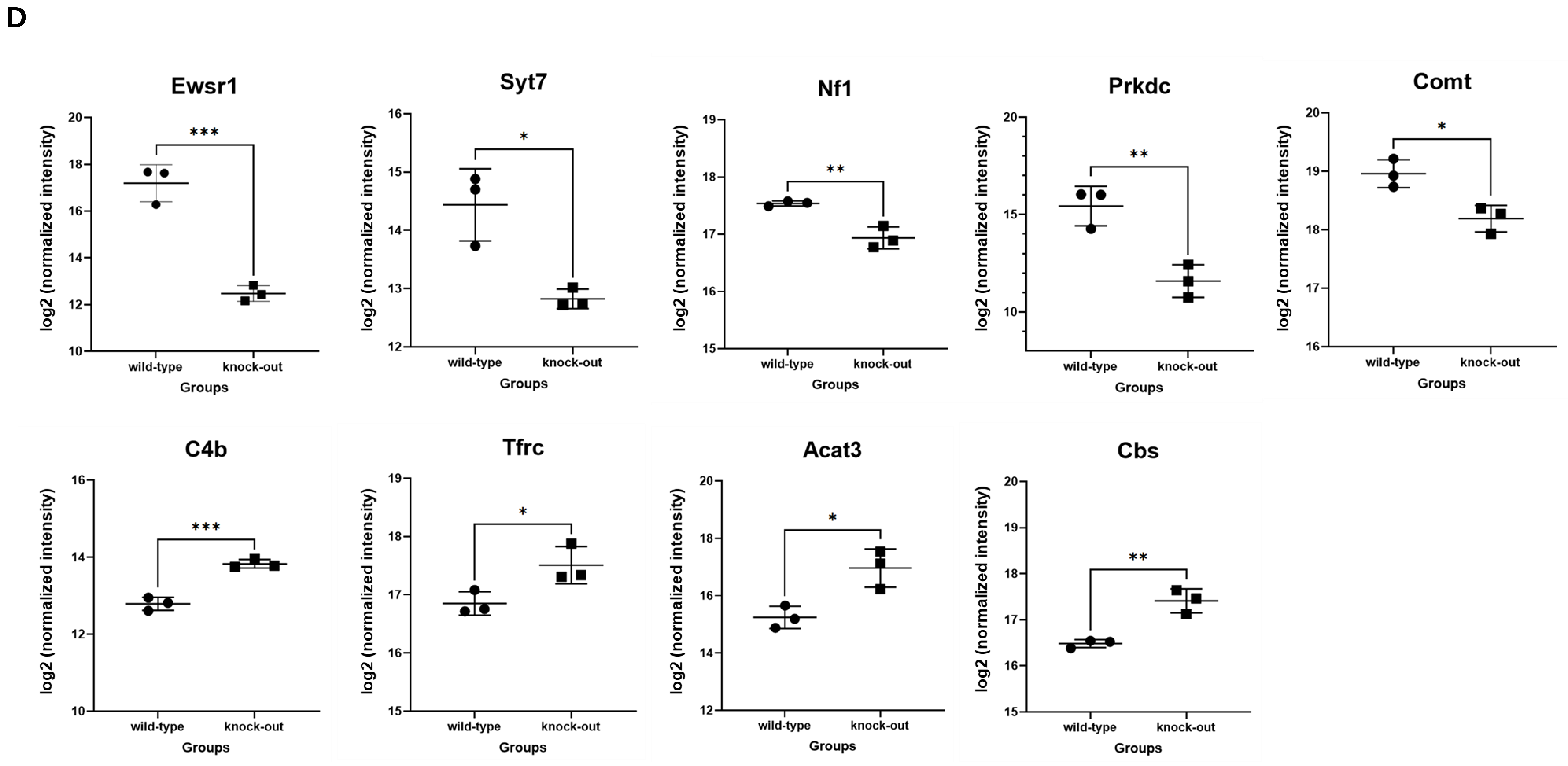

Supplementary Figure S5. Data-independent acquisition (DIA) proteomics. (A) Quantitative correlation plot in log2(intensity) with R-squared (R^2) value. (B) Coefficient of variance of each protein in WT and KO group. (C) Correlation plot comparing Log_2fold-change(FC) values of significantly differentially expressed targets identified in TMT and DIA datasets (D) Box plots of nine proteins (Ewsr1, Syt7, Nf1, Prkdc, Comt, C4b, Tfrc, Acat3, and Cbs). The asterisk indicates the p-value of the statistical test (* $P < 0.05$;** $P < 0.01$; *** $P < 0.001$)

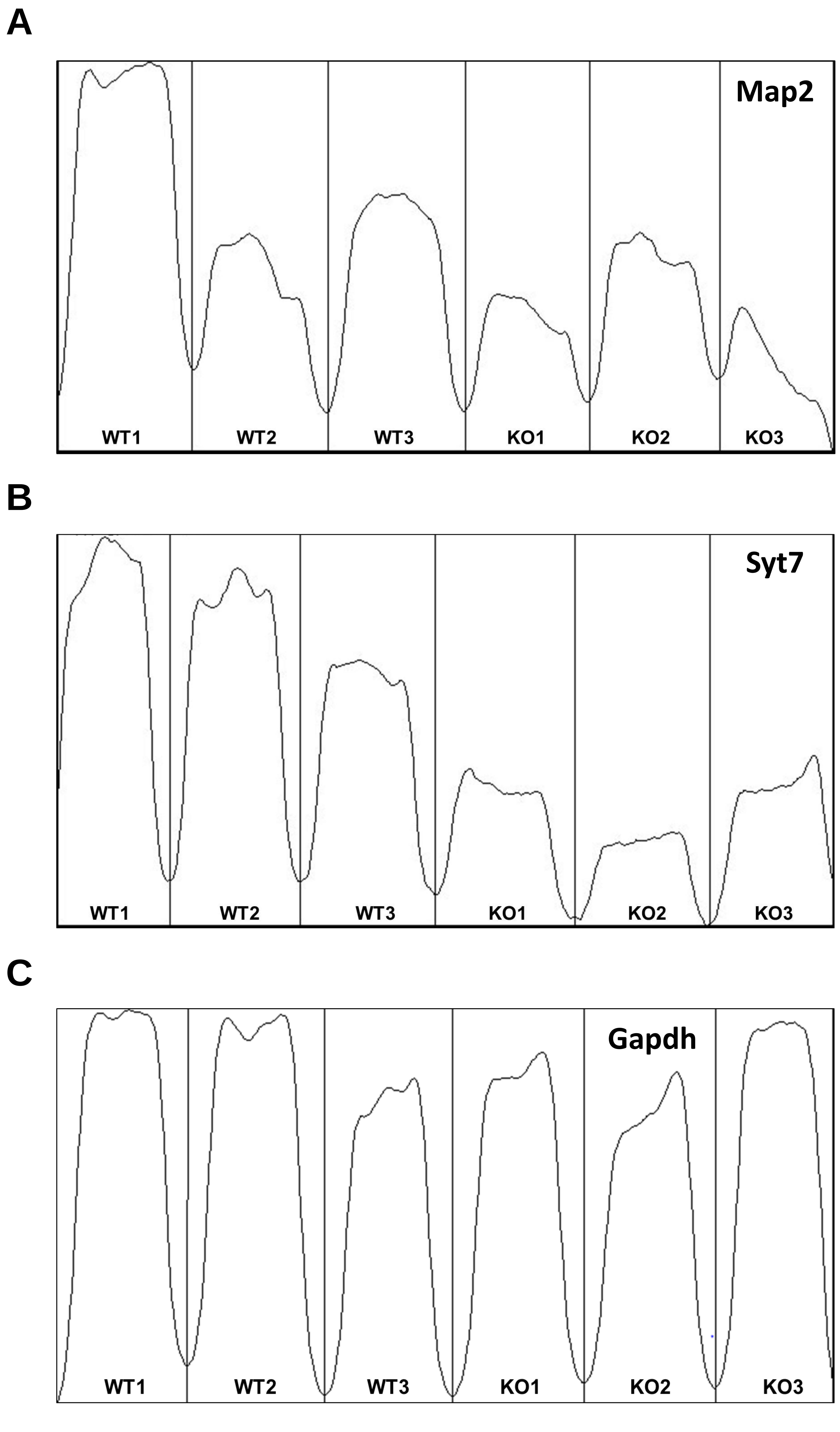

Supplementary Figure S6. Densitometry plots for the western-blot analysis.

(A) Map2 (B) Syt7 (C) Gapdh

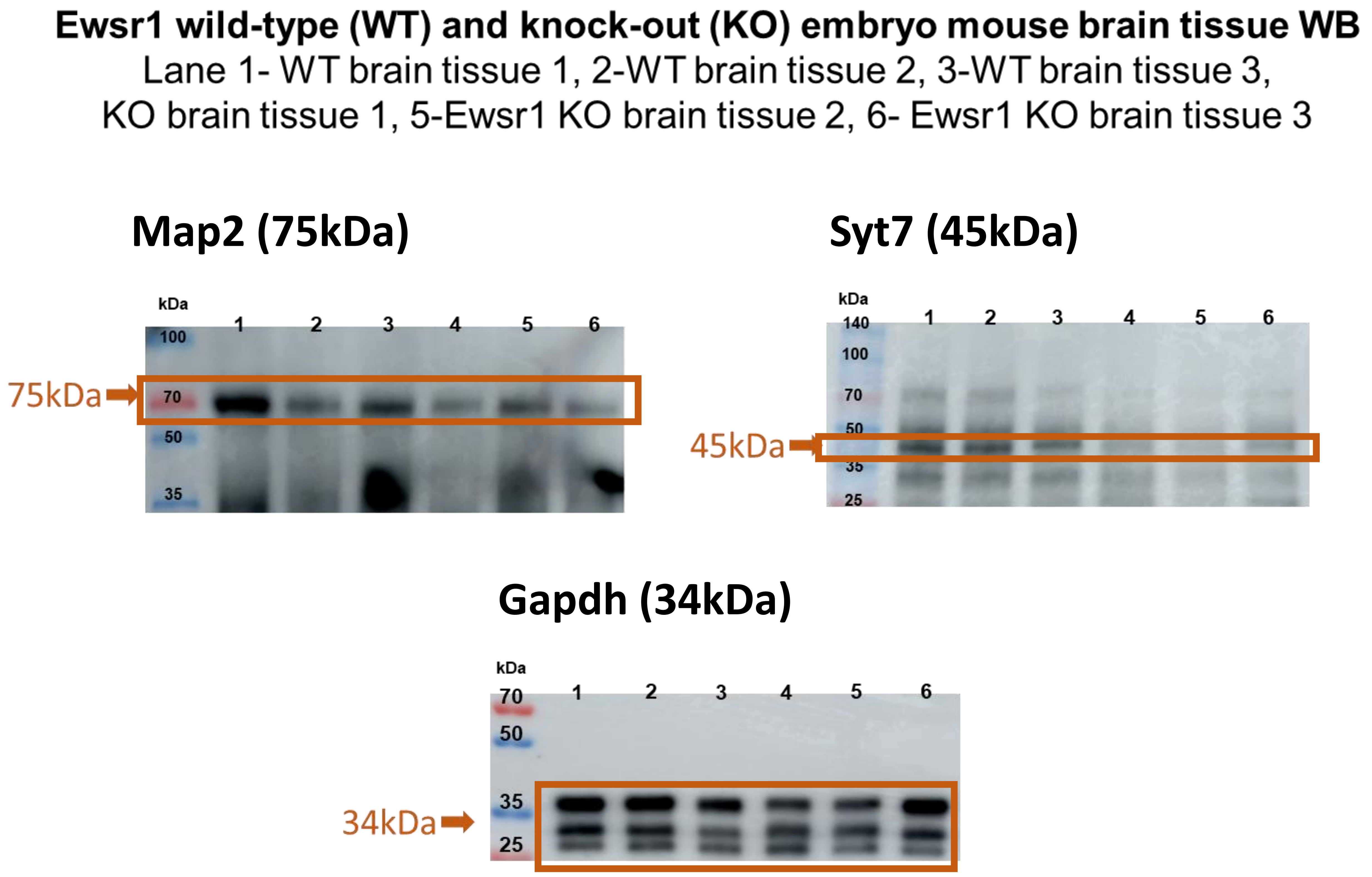

Supplementary Figure S7. Raw data of western blot analysis. Lanes 1-3 represent brain samples from wild-type mouse 1,2, and 3, respectively. Lane 4-6 represent brain samples from Ewsr1 knockout mouse 1,2, and 3, respectively
